# Transition from traveling fronts to diffusion-limited growth in expanding populations

**Published:** 2026-02-12

**Authors:** Louis Brezin, Kyle J. Shaffer, Kirill S. Korolev

**Affiliations:** Department of Physics and Graduate Program in Bioinformatics, Boston University, Boston, Massachusetts 02215, USA; Gettysburg College, Gettysburg, Pennsylvania 17325, USA; Department of Physics, Graduate Program in Bioinformatics, and Biological Design Center, Boston University, Boston, Massachusetts 02215, USA

## Abstract

Reaction-diffusion equations describe various spatially extended processes that unfold as traveling fronts moving at constant velocity. We introduce and solve analytically a model that, besides such fronts, supports solutions advancing as the square root of time. These sublinear fronts preserve an invariant shape, with an effective diffusion constant that diverges at the transition to linear spreading. The model applies to dense cellular aggregates of nonmotile cells consuming a diffusible nutrient. The sublinear spread results from biomass redistribution slowing due to nutrient depletion, a phenomenon supported experimentally but often neglected. Our results provide a potential explanation for the linear rather than quadratic increase of colony area with time, which has been observed for many microbes.

When non-motile cells grow, they form dense aggregates such as healthy tissues, tumors, biofilms, microbial mats, and colonies. The growth dynamics of such aggregates influence diverse phenomena, including disease onset and progression, agricultural productivity, geochemical cycles, and the integrity of human-built infrastructure [1–8]. Consequently, understanding these dynamics has been a focus of extensive research, employing both detailed application-specific models and simpler phenomenological frameworks aimed at uncovering general principles of population growth [9–23].

Among these approaches, reaction-diffusion equations have emerged as the dominant modeling paradigm, because they effectively incorporate nutrient diffusion, cellular growth and motility, mechanical interactions, and other key processes. Theoretical predictions has been most thoroughly tested in the context of microbial colonies due to their accessibility for quantitative measurement and manipulation. In particular, reaction-diffusion models have successfully explained complex pattern formations [10–13, 15] and—perhaps most notably—the observed nearly constant expansion velocity of microbial colonies [24–27]. This constant front velocity is a striking prediction resulting from the interplay of diffusive transport and exponential growth.

Recent theoretical work has focused on how various biophysical processes, especially mechanical interactions, influence expansion velocity [17–23]. However, an increasing number of experiments suggest that the commonly assumed linear growth is not universal. In particular, many organisms under diverse growth conditions exhibit sublinear, power-law growth with an exponent close to one-half [26, 28–30]. Here, we demonstrate that these experimental observations can be reconciled within the standard reaction-diffusion framework by incorporating the experimentally motivated dependence of biomass redistribution on nutrient concentration—a factor largely overlooked in previous models.

Although there are a great number of reaction-diffusion models of colony growth, they typically fall into one of three classes. The first class includes various generalizations of the Fisher-Kolmogorov-Petrovsky-Piskunov (FKPP) equation [31–33]:

(1) $$\frac{\partial b}{\partial t}=D_{s}\nabla^{2}b+rb\left(1-\frac{b}{K}\right).$$

Here, the growth rate of biomass b is approximated by the standard logistic curve, which consists of exponential growth at low b and saturation at carrying capacity K. The value of K is set by the initial nutrient concentration, which is not modeled explicitly. The motility is assumed to follow a random-walk-like pattern, with the effective diffusion constant given by $D_{s}$. This classic equation was the first model of reaction-diffusion waves in population biology and motivated numerous subsequent studies in various fields [33, 34]. It predicts invariant traveling fronts moving with velocity $v=2\sqrt{D_{s}r}$ and an exponentially decreasing population density ahead of the wave. These predictions have been confirmed in many experimental and observational studies [25, 33, 35–38], but only with motile organisms, e.g., bacteria swimming in very thin agar. In dense microbial colonies, the outward motion of cells is not diffusive, and population density abruptly drops to zero instead of showing a more gradual exponential decrease [9, 17–23, 26].

To capture the sharp drop of the biomass at the front, density dependence was introduced in the diffusion term of the FKPP equation [33, 34]:

(2) $$\frac{\partial b}{\partial t}=D_{p}\nabla \cdot (b\nabla b)+rb\left(1-\frac{b}{K}\right),$$

where the new parameter $D_{p}$ quantifies the emergent cooperative motility of the cells and could depend on many factors such as the agar concentration, surfactant production, and cell rigidity. Phenomenologically, the nonlinear diffusion could be explained by collective motion due to the repeated rearrangements of cells within the colony as they push against each other. Alternatively, the nonlinear diffusion can be derived from a hydrodynamic model that involves mechanical compression due to growth, friction with the substrate, and the flow of the biomass in response to mechanical forces (see the Supplemental Material [39]). The front velocity in this model equals $\sqrt{D_{p}r/2}$, and the population density vanishes linearly near the colony edge [34, 40–42]; power law decay is also possible for slightly different models [39].

Although Eq. (2) recapitulated the growth of circular colonies reasonably well, it could not reproduce two essential aspects of colony growth. First, colonies stop growing well before reaching the edge of the Petri dish, and, second, colonies exhibit non-circular (rough or branched) morphologies at low nutrient and high agar concentrations [10–12, 29]. Both of these observations can be explained by nutrient limitation [9–12], which is introduced in the third class of models:

(3) $$\begin{array}{l} \frac{\partial b}{\partial t}=D_{p}\nabla \cdot \left(b\nabla b\right)+\gamma bn,\\ \frac{\partial n}{\partial t}=D_{n}\nabla^{2}n-\gamma bn. \end{array}$$

Here, n is the concentration of the growth-limiting nutrient, $D_{n}$ is its diffusion constant, and γ is the nutrient consumption rate. For simplicity, we assume that the biomass is measured in units such that one unit of nutrient produces one unit of biomass. We also neglect the metabolic cost of maintenance and the saturation of the nutrient uptake at high n. These and other complications can be included, but they lead to similar dynamics; see the Supplemental Material [39].

Typically, small molecules diffuse much faster than the biomass. As a result, the nutrients are depleted in a region of about $D_{n}/v$ ahead of the front, and the nutrient concentration within the colony is quite low. In fact, it decreases with $D_{n}$, and the expansion velocity scales as $D_{p}/\sqrt{D_{n}}$ in contrast to the other two models discussed above [9].

Despite the great success of Eq. (3) in capturing many properties of microbial colonies, it only describes traveling fronts moving with a constant velocity just like Eqs. (1) and (2) [9–12]; see also Fig. S2. While the radius of certain microbial colonies indeed grows linearly with time, it is the area that increases linearly in other experiments with seemingly similar organisms and growth conditions [26, 28–30, 43].

The most extensive evidence for sublinear growth comes from the high-throughput screen of microbial growth patterns by Ernebjerg and Kishony [28] who measured how the radius of the colony increased with time for nearly five hundred colonies from soil isolates. The majority of the growth patterns were well described by a sublinear power law with exponent values clustered around 0.5. That is, the area of the colony rather than its diameter increased linearly with time for the majority of the colonies. The sublinear growth of the radius is often attributed to nutrient exhaustion, drying of the agar surface, or other experimental artifacts. While such mechanism should certainly be explored, it is unlikely that they explain these and other observations of a clearly linear increase of the area with time. In Ernebjerg and Kishony’s experiments, colonies had very variable lag times and achieved variable final sizes, which means that they exhibited power law growth despite different stages of agar drying and varying degrees of nutrient depletion.

The robustness against experimental artifacts is further confirmed by the studies of *Bacillus subtilis* and *Saccharomyces cerevisiae,*, two model organism whose growth has been carefully characterized by different experimental groups [29, 30, 43]. Their results invariably show a very clean power law that starts after about ten hours of growth and continues for several days until either the experiment is terminated or the growth stops due to nutrient depletion.

To understand the origin of this common, but rarely characterized growth pattern, we carefully examined the assumptions behind Eq. (3). Among all of the simplifications in this model, the most questionable assumption is the functional form of the biomass motility because it has not been carefully quantified. In fact, existing experimental data strongly suggests that the rate of biomass redistribution depends, perhaps indirectly, on the nutrient concentration. The main evidence comes from experiments with two identical strains labeled by different fluorescent markers [27, 44, 45]. As the colonies expand, demographic fluctuations lead to local fixation of one of the strains and the establishment of monoclonal sectors. The boundaries between these sectors are dynamic at the colony edge, where cells are actively growing and the nutrient concentration is high, but they are frozen in the colony bulk. Thus, biomass redistribution requires active growth, which in turn requires nutrients. Further supporting evidence comes from competition experiments with strains that grow at different rates [46]. In this study, it was found that the differences in expansion velocities are proportional to the differences in the growth rates in liquid culture. This can be reconciled with the predictions of Eqs. (1), (2), and (3) only when the biomass motility depends on the growth rate; otherwise, the expected dependence is $v\propto \sqrt{r}$ and $v\propto \sqrt{\gamma n}$ respectively, which does not match the experimental data.

Based on these observations, we modified Eq. (3) to capture the link between motility and growth as follows

(4) $$\begin{array}{l} \frac{\partial b}{\partial t}=D_{b}\nabla \cdot (bn\nabla b)+\gamma bn,\\ \frac{\partial n}{\partial t}=D_{n}\nabla^{2}n-\gamma bn. \end{array}$$

Note that the effective diffusion coefficient has the same dependence on b and n as the growth term; in other words, we could say that the motility rate is proportional to the net growth rate. In the Supplemental Material, we discuss alternative formulations of this model and provide a derivation based on the balance of mechanical forces within the colony [39]. The only difference between this derivation and that of Eq. (2) is that we account not only for cell compression, but also for active stresses generated by colony growth.

To simplify the analysis, we nondimensionalize our model by measuring b and n in the units of the initial nutrient concentration $n_{0}$, time in units of $1/\left(\gamma n_{0}\right)$, and spatial positions in the units of $\sqrt{D_{n}/\left(\gamma n_{0}\right)}$. This transformation sets $n_{0}$ and all the coefficients in Eq. (4) to unity except for the biomass diffusivity, which becomes

(5) $$D=\frac{D_{b}n_{0}^{2}}{D_{n}}.$$

In the following, we use the nondimensionalized formulation without a change in notation. Note that the dimensional velocities are given by the nondimenionalized velocity v times the “nutrient velocity” given by $v_{n}=\sqrt{D_{n}\gamma n_{0}}$.

Using numerical simulations [39], we examined the expansion of the biomass in d spatial dimensions. We primarily focus on the expansions in narrow channels d=1 and on the surface of a Petri dish d=2, but other values of d are discussed in the Supplemental Material [39]. To simplify the calculations, our analytical and numerical analysis is focused on radially symmetric solutions. Thus, for d>1, we assume that either growth instabilities do not occur or they are suppressed by large surface tension or other factors. This is a reasonable assumption because experiments show that mutations that reduce cellular adhesion result in perfectly circular colonies without any signs of instabilities even at low nutrient and high agar concentrations [29], i.e., in the regime where many organisms produce rough colonies with finger-like protrusion or branches. In the following, we use the radial coordinate r when we are describing the results for d>1 and the linear coordinate x for the results specific to d=1.

Figure 1) shows our key result: different growth regimes for high and low D. For large values of D, the radius of the colony increased linearly with time, and the solution behaved as a standard reaction-diffusion front. Below a critical value of biomass diffusivity $D_{c}\approx 1$, the nature of the solution changed. The radius of the colony increased only as $t^{1/2}$. Despite this slower growth, the spatial profile of the biomass density remained invariant in the co-moving reference frame, similar to a regular traveling front.

First, we tested that Eq. (4) indeed admits solutions of the form b(x−vt) and n(x−vt). Upon substituting the traveling-front ansatz into the equation, we solved the resulting ordinary differential equations both numerically using the shooting method and analytically by making certain approximations; see the Supplemental Material [39]. Both calculations confirmed that traveling front solutions exist only for $D\;>\;D_{c}$ and showed the same behavior as the solutions of the time-dependent problem (Fig. 2).

The analytical solution for v(D) provides approximate, but very simple summary of our results:

(6) $$v=\frac{D\;-\;1}{\sqrt{D}}.$$

In agreement with simulations, v∝D for large D, and the velocity vanishes at $D_{c}\;=\;1$. The former scaling is the same as for Eq. (2) because there is no nutrient limitation in this regime. The large D behavior is, however, not relevant for microbial colonies for which v<1 (i.e., ${v\;<\;v}_{n}$ in dimensional units) [17, 27, 46]. Note that our results for $D\;>\;D_{c}$ do not depend on the number of spatial dimensions because the traveling front solution emerges after a short transient when the radius of the colony is much larger than the thickness of the growth front [33, 39].

Second, we examined the solutions that exhibit the square-root growth. Although both n and b moved forward as $t^{1/2}$, only the biomass profile remain invariant in time, just as for $D\;>\;D_{c}$. The nutrient profile was not a traveling wave. Instead, the nutrient concentration at the edge of the colony decreased as $t^{1/2}$, and the region of nutrient depletion ahead of the colony increases as $t^{1/2}$; see Figs. S6 and S7. Hence, the length scale on which the nutrient varies becomes much larger than that on which the biomass varies, and we can simplify the problem by assuming that b(t,r) is a moving Heaviside step function:

(7) $$b(t,r)=H\theta \left(r-r_{e}(t)\right),$$

where $r_{e}(t)$ is the position of the colony edge, and H is the biomass density within the colony, which reflects the height or thickness of an actual three-dimensional colony. The motion of the colony edge is then given by nutrient flux into the colony since nutrients are converted into biomass without loss in Eq. (4):

(8) $$H\frac{dr_{e}}{dt}={\left.\frac{\partial n}{\partial r}\right|}_{r=r_{e}}.$$

To the leading order, the equation for n(t,r) becomes a simple diffusion equation with an absorbing (Dirichlet) boundary condition at $r=r_{e}(t)$.

The simplified problem can be solved by the standard methods; see the Supplemental Material [39], and the solution reads

(9) $$r_{e}\left(t\right)=2\varkappa \sqrt{t},$$

where ϰ is specified, in terms of H, by the following equation

(10) $$H^{-1}\;=2\varkappa^{d}e^{\varkappa^{2}}\int_{\varkappa }^{+\infty }p^{1-d}e^{-p^{2}}dp\;\;=\left\{\begin{array}{c} \sqrt{\pi }\varkappa e^{\varkappa^{2}}erfc(\varkappa )d=1\\ \varkappa^{2}e^{\varkappa^{2}}E_{1}\left(\varkappa^{2}\right)d=2 \end{array}\right.$$

Here, erfc(y) and $E_{1}(y)$ are the complementary error function and exponential integral respectively. The limiting behavior for small and large ϰ is discussed in the Supplementary Material [39]. Briefly, H→1 from above for ϰ→+∞, and H→+∞ when ϰ→0. That is, colonies that expand more slowly are thicker.

We can test Eq. (10) by obtaining H and ϰ from simulations for various values of D, and then comparing the observed values of H to the values of H predicted by Eq. (10). This comparison is shown in Fig. 3b, and the agreement between the analytic and the numerical solutions is excellent.

Our simplified model determines the behavior of b and n, but it contains one unknown parameter, the height of the colony H. In the full model, H must be a function of D, which is absent in the simplified model. Naturally, we expect that H is large at small D, when the colony barely moves, and the biomass must accumulate in the vertical direction. In the opposite limit of $D\to D_{c}$, we expect H→1, since the solution should approach the traveling front limit, for which it is easy to show that $H=n_{0}$. In the Supplemental Material [39], we derive an approximate expression for H(D), which is given by

(11) $$H=\frac{1}{\sqrt{D}}.$$

Figure 3 confirms that this prediction captures the qualitative behavior of ϰ(D) and H(D) extremely well. In dimensional units, $H=\sqrt{D_{n}/D_{b}}$. Thus, the height of the colony scales with $n_{0}$ for $D>D_{c}$, but, in the square-root regime, it is independent of the nutrient concentration. Instead, it is controlled by $D_{b}$, with greater motility leading to thinner colonies.

The analysis of the square-root growth is now complete because we can obtain ϰ(D) by combining Eqs. (10) and (11); see Supplemental Material for a detailed calculation [39]. When D is close to $D_{c}$, we find that $\varkappa \approx \sqrt{D_{n}d/(2(1-\sqrt{D_{b}n_{0}^{2}/D_{n}}))}$ in the dimensional units. Thus, ϰ diverges prior to the transition to traveling fronts, and the expansions are slightly faster in d=2 compared to d=1. For small D, the effect of expansion geometry is more dramatic.

For d=1, we find that $\varkappa =n_{0}\sqrt{D_{b}/\pi }$; i.e., the rate of colony expansion is independent of $D_{n}$ even though the growth is diffusion limited. The total biomass in the colony, $B(t)=2n_{0}\sqrt{D_{n}t/\pi }$, which equals the amount of nutrients absorbed by a stationary colony during time t. Therefore, small $D_{b}$ speeds up colony expansion, but does not lead to greater biomass accumulation because $D_{b}$ also reduces H. We then expect no selective advantage of larger $D_{b}$ when the biomass motility is weak. In contrast, our results above show that both ϰ and B(t) dramatically increase with $D_{b}$ near the transition to traveling fronts, which indicates that the selective pressure on $D_{b}$ could vary substantially across different growth environments.

For d=2, we find that expansions proceed faster than one dimension, and $\varkappa =D_{n}^{1/4}D_{b}^{1/4}n_{0}^{1/2}/\sqrt{ln\left(D_{b}n_{0}^{2}/D_{n}\right)/2}$, so both $D_{n}$ and $D_{b}$ affect the rate of colony growth. In contrast, the accumulation of the biomass depends on $D_{b}$ only logarithmically: $B(t)=-16\pi n_{0}D_{n}t/ln\left(D_{b}n_{0}^{2}/D_{n}\right)$. Experimentally, the easiest quantity to vary is $n_{0}$, and our results predict that $\varkappa \propto n_{0}$ in narrow channels (d=1) while, up to logarithmic corrections, $\varkappa \propto \sqrt{n_{0}}$ for circular colonies (d=2). Thus, the growth geometry controls not only the rate of colony growth, but also its dependence on the nutrient concentration.

In summary, we modified the standard model of colony growth Eq. (3) to make it consistent with the experiments reporting no biomass motion in regions without growth. We achieved this by making the rate of biomass redistribution proportional to the nutrient concentration, which effectively accounts for the active mechanical stresses within the growing colony. Unlike most reaction-diffusion models, Eq. (4) predicts two regimes of colony expansion: one with the radius of the colony increasing linearly with time and one with the square-root increase. The linear regime is described by the standard framework of traveling fronts, and we determined how the velocity of the expansion depends on model parameters. The square-root regime is different. The shape of the biomass profile remains invariant in time, just as in the traveling fronts regime, but the nutrient profile becomes progressively wider and more depleted.

At first sight, it is not surprising that growth dynamics limited by nutrient diffusion lead to the $t^{1/2}$ scaling of the colony radius with time [17, 47]. We note, however, that the rate of this square-root growth could be much higher than that of a stationary colony passively absorbing nutrients. This greater expansion rate results from the nontrivial coupling between the spatial advance of the colony and the rate of nutrient acquisition summarized by Eq. (10).

Our model and its predictions are relatively straightforward to test experimentally. To characterize colony growth, we introduced only a handful of parameters: the initial nutrient concentration, the nutrient consumption rate, the nutrient diffusion constant, and the biomass motility. All but the last parameter can be easily measured experimentally. Thus, we have only one parameter to fit all possible observations, which include the dependence of the radius on time, colony thickness, nutrient profiles, and transitions between different growth regimes as a function of D and expansion geometry (narrow channels vs. a Petri dish). In fact, studying colony thickness and growth dynamics as a function of $n_{0}$ in different geometries appears to be the most straightforward way to validate our results.

In closing, let us speculate that typical microbial colonies could be near the transition between the linear and the square-root growth regimes, which would explain why certain experiments report the linear growth of the radius while others report the linear increase of the area of microbial colonies without a major shift in the colony shape. In the future, we hope to explore whether the square root regime has any marked effects on the ecological and evolutionary dynamics within the colonies.

## Supplementary Material

Supplement 1

## Figures and Tables

**FIG. 1.**
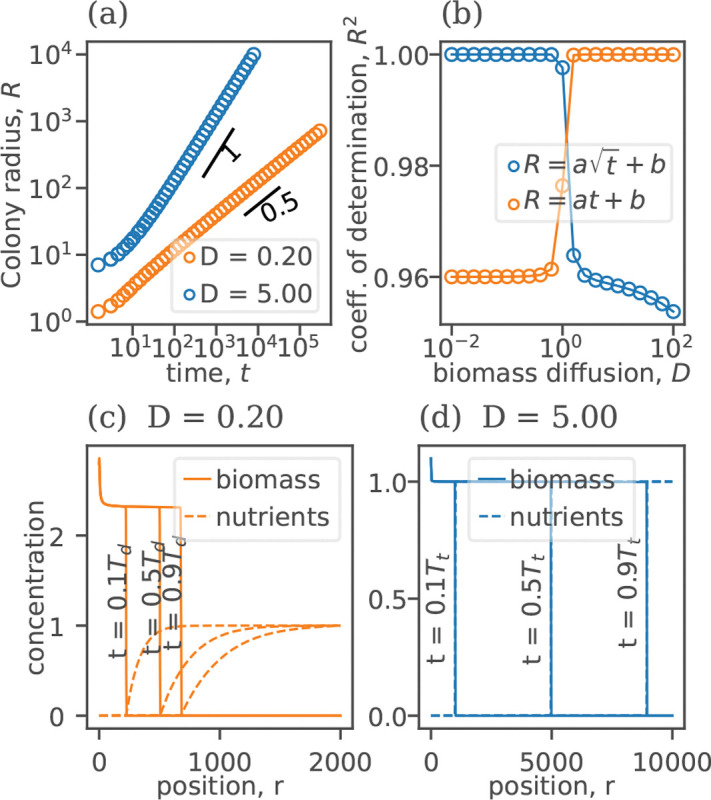
(color online). Transition between traveling fronts and diffusion-limited growth. (a) Equation (4) supports two types of solutions with the colony radius R increasing either linearly with time or as a square root of time. (b) The coefficient of determination ($R^{2}$) indicates that $R\propto \sqrt{t}$ for $D<D_{c}$, and R∝t for $D>D_{c}$. Examples of biomass and nutrient profiles in the radial direction, at 10%, 50%, and 90% of the time preceding nutrient depletion at the end of the simulation box are shown in (c) for $D<D_{c}$ and in (d) for $D>D_{c}$.

**FIG. 2.**
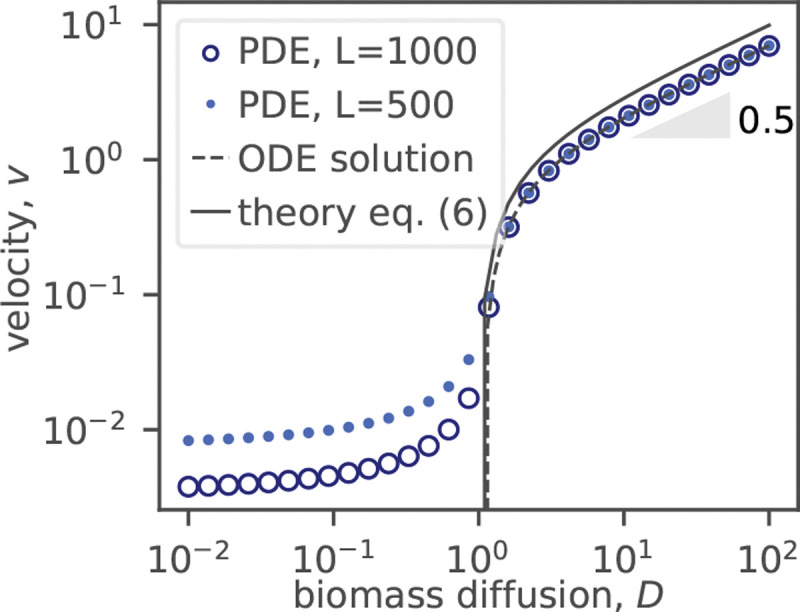
(color online). Velocity of the traveling front solutions. Circles show the velocities obtained by performing a linear fit on R(t), determined by solving Eq. (4) (PDE). These calculations were performed at two systems sizes. The disagreement between them indicates that a traveling front solution does not exist. The dashed line shows the results of the shooting method (ODE), and the solid line is the analytical approximation given by Eq. (6).

**FIG. 3.**
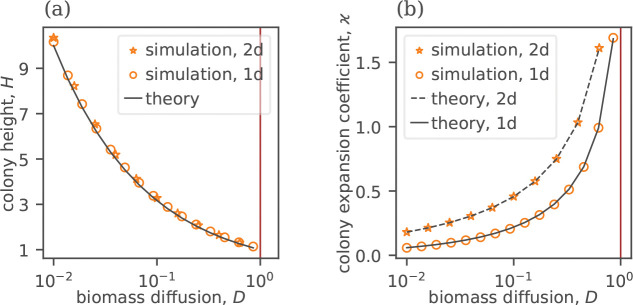
Biomass diffusivity controls the height and expansion rate of microbial colonies in the regime of square-root growth, as demonstrated for simulations in one and two dimensions. (a) The colony height, defined as the maximal concentration near the edge of the colony, diverges for D→0, as expected for a stationary colony, and it approaches 1 for $D\to D_{c}$, as expected for a traveling front solution. The solid line shows the analytical prediction given by Eq. (11). (b) The rate of colony expansion, quantified by $\varkappa =r_{e}/(2\sqrt{t})$, increases with D. It approaches zero for D=0 and diverges at $D\to D_{c}$. Theoretical predictions based on Eq. (10) are shown with a solid line for 1d and a dashed line for 2d, using numerically obtained values of H.
