# Supplementary material for: Transition from traveling fronts to diffusion-limited growth in expanding populations
Source: ArXiv. 2026 Feb 12:arXiv:2602.11540v1. Preprint. [Version 1] (PMC12919227)
Supplement: Supplement 1 [file NIHPP2602.11540v1-supplement-1.pdf]

SUPPLEMENTARY MATERIAL

This Supplemental Material contains detailed derivations and supporting data for the results reported in the main text. First, we show how the reaction-diffusion equations presented in the main text can be derived from a more detailed description of mechanical interactions between the cells, as well as between the cells and the substrate. We also discuss possible generalizations and alternative forms of the models described in the main text. The next two sections contain the analysis of the traveling fronts and square-root solutions of Eq. (4) in the main text. The fourth section provides details of the numerical methods, and the final section contains figures that provide additional details for our main results.

Mathematical models

Mechanics of colony growth
The motion of cells within the colony can be described by a velocity field $\boldsymbol{v}$. The biomass dynamics is then governed by

$$\frac{\partial b}{\partial t} = -\boldsymbol{\nabla} \cdot (b\boldsymbol{v}) + r(b, n)b, \tag{S1}$$

which combines advection with velocity $\boldsymbol{v}$ and growth with the growth rate $r(b, n)$. Assuming that the mechanical properties of the colony are isotropic, the velocity field can be determined from the pressure field p by balancing the elastic and frictional forces:

$$-\boldsymbol{\nabla} p - \zeta_0(b, n) - \zeta_1(b, n)\boldsymbol{v} = 0, \tag{S2}$$

where ζ_0 accounts for static friction and ζ_1 for viscous friction. The dependence of viscosity on v is neglected, and we also neglect the contribution of static friction in the following [17].

To close this system of equations, we need a constitutive relationship between p, b, and n. The standard assumption that stress is linear in strain can be expressed as

$$p = p_0 + \kappa(b, n)b, \tag{S3}$$

where p_0 is a constant, and κ is a measure of compressibility. Next, we express p in terms of b and substitute the result into Eq. (S2) to find $\boldsymbol{v}$:

$$\boldsymbol{v} = -\frac{\boldsymbol{\nabla}(\kappa b)}{\zeta_1}. \tag{S4}$$

We are now in a position to obtain the final reaction-diffusion equation that involves only b and n by substituting Eq. (S4) into Eq. (S1). The result reads

$$\frac{\partial b}{\partial t} = \boldsymbol{\nabla} \cdot \left(b\frac{\boldsymbol{\nabla}(\kappa b)}{\zeta_1} \right) + r(b, n)b. \tag{S5}$$

From this equation, we can obtain all the different functional forms of the biomass redistribution term discussed in the main text. If we assume that κ depends neither on n nor on b and that ζ_1 is a linear function of b, i.e., $\zeta_1 = \zeta_1' b$, then we obtain the classic FKPP equation with $D_s = \kappa/\zeta_1'$. The first assumption is a common approximation for an elastic material, and the second assumption posits that the frictional force is directly proportional to the number of cells, which is the case for swimming motility.

The population pressure model in Eqs. (2) and (3) of the main text is obtained when both κ and ζ_1 are assumed to be constant. The latter assumption is equivalent to saying that, for a dense collection of cells, the frictional force with the substrate does not depend on the colony height.

If we assume that ζ_1 is constant and $\kappa = \kappa' n$, then we obtain a model very similar to Eq. (4) in the main text:

$$\frac{\partial b}{\partial t} = \frac{\kappa'}{\zeta_1}\boldsymbol{\nabla}(b\boldsymbol{\nabla}(nb)) + r(b,n)b. \tag{S6}$$

Although different in general, these equations are approximately the same at the colony edge, which is the only region relevant to the growth dynamics. Ahead of the edge, $b = 0$, and, behind the edge, the biomass approaches its limiting value 1 or H, so its dynamics is not relevant and all of the terms in the biomass equations are negligible. At the colony edge, b varies much more rapidly than n, at least for $D \ll 1$ (see fig. S5), so the gradients of n can be neglected compared to the gradients of b, i.e., we can approximately treat n as a constant. Therefore, we expect the same qualitative behavior as in the model discussed in the main text. Note that the linear dependence of κ on n is an unusual assumption. Potentially, it can be justified by considering the fact the pressure within the colony reflects both static compression and a dynamic component due to cell growth, which increases with the nutrient concentration.

Finally, our exact model is obtained by assuming that $\zeta_1 = \tilde{\zeta}/n$ and κ is a constant. Here, the key assumption is that the activity of growth reduces the friction with the substrate. We are not aware of any evidence supporting such an assumption, and we believe that the arguments leading to Eq. (S6) provide a better justification for Eq. (4) in the main text. It is also clear that the type of reasoning outlined above could lead to more general models of the following form:

$$\frac{\partial b}{\partial t} = \boldsymbol{\nabla}\cdot\left(D_1 b^{\beta} n \boldsymbol{\nabla} b + D_2 b^{\beta'}\boldsymbol{\nabla} n\right) + r(b,n)b. \tag{S7}$$

We briefly explored a few of such models (see Fig. S1) and found the same qualitative dynamics as reported in the main text.

Models of nutrient consumption

In the main text, we considered a simple expression for biomass growth and nutrient consumption given by γbn. The actual dynamics could include a number of complications. Two most important ones are discussed below.

First, at high nutrient concentration, both the consumption and the growth rate should saturate to a value independent of n; we will denote it by r to make the connection with the FKPP equation. Then the Monod growth kinetics can be expressed as

$$r(b,n) = \frac{\gamma n}{1+\gamma n/r}, \tag{S8}$$

where γ and r are the same as in the main text. In particular, r reflects the maximum growth rate ($\mu_{\max}$ in the Monod equation) and the half-saturation constant is r/γ.

The nutrient consumption could be different from $r(b,n)$ if the yield depends on the nutrient concentration. A simple Michaelis-Menten model would lead to

$$\frac{\partial n}{\partial t} = D_n\boldsymbol{\nabla}^2 n - \frac{\gamma nb}{1+\gamma n/r'}, \tag{S9}$$

with $r' \geq r$. For large D, we still expect traveling fronts with velocities that scale as $\sqrt{D_b}$ when $D_b \to +\infty$ because D_n is irrelevant and D_b is the only parameter containing the dimension of length. The dependence of v on n_0 will be different depending on the values of γ and r. For small D, the nutrient concentration at the edge of the colony decreases with time and eventually falls well below the half-saturation constant. At this point, the more complex models based on the Monod and Michaelis-Menten kinetics reduce to the simpler terms studied in the main text. Therefore, we expect no major differences for small D_b near or below the transition to square-root growth.

Second, at very low nutrient concentrations, one has to account for the metabolic cost of cell maintenance because it becomes a significant fraction of total energy expenditure when the growth rates are low. A simple, if a little crude, way to account for these effects is to introduce a minimal nutrient concentration n_m required for growth:

$$r(b,n) = \frac{\gamma n}{1+\gamma n/r}\theta(n-n_m) \tag{S10}$$

$$\frac{\partial n}{\partial t} = D_n\boldsymbol{\nabla}^2 n - \frac{\gamma nb}{1+\gamma n/r}. \tag{S11}$$

This modification makes no appreciable effect on the dynamics of traveling fronts except in the immediate vicinity of the transition when v is small. Indeed, we expect that $n_e \approx 1 - 1/D$; see Eq. (S25), so n_e stays above n_c and the maintenance cost has no effect on the growth term at the front. Behind the front, the maintenance cost reduces the biomass concentration in the colony bulk below n_0.

The maintenance cost, however, completely destroys the asymptotic $t^{1/2}$ growth because the colony stops growing altogether once the nutrient concentration falls below n_c at the colony edge. Since $n_e \propto t^{-1/2}$, this growth arrest is inevitable. That said, the time required to deplete the nutrients below n_c could be quite long, and the intermediate behavior is given by the square-root growth as before. In other words, the $t^{1/2}$ behavior for growth becomes an intermediate asymptotic. This behavior is illustrated in Fig. S1, in the limit of large r.

Traveling-front solutions

By definition, traveling wave solutions are plane waves, i.e. $b(t, \boldsymbol{r}) = b(x - vt)$ for waves traveling in the positive x-direction. When growth instabilities are suppressed by internal dynamics, surface tension, or other mechanisms, the planar traveling fronts are also good approximations for growing circular colonies. Indeed, the width of traveling fronts (and the nutrient depletion layer) are finite and, as we show below, are typically small; therefore, the effect of circular geometry should decrease as the inverse of the colony radius. For the FKPP equation, the effects of front curvature are discussed in Ref. [33]. We limit our discussion of traveling front solution to planar wave fronts, which are effectively one-dimensional.

Properties of the solutions and the shooting method
In this section, we analyze the behavior of the traveling front solutions of Eq. (4) near the colony edge and deep in the colony bulk. This analysis provides the initial and final conditions for the shooting method to determine the front profiles and velocities numerically. We also derive the analytical approximation for $v(D)$ given by Eq. (6) by matching the initial and final conditions.

We begin by stating the reaction-diffusion model in the nondimensionalized variables:

$$\begin{aligned} \frac{\partial b}{\partial t} &= D\frac{\partial}{\partial x}\left(bn\frac{\partial b}{\partial x}\right) + bn, \\ \frac{\partial n}{\partial t} &= \frac{\partial^2 n}{\partial x^2} - bn. \end{aligned} \tag{S12}$$

with the initial nutrient concentration equal to 1. We then substitute the traveling wave ansatz into this partial differential equation. Specifically, we assume that b and n are functions of z only, with $z = x - vt$. The resulting system of ordinary differential equations reads

$$\begin{aligned} &D(bnb')' + vb' + bn = 0, \\ &n'' + vn' - bn = 0, \end{aligned} \tag{S13}$$

where primes denote derivatives with respect to z. To solve these equations, we also need the boundary conditions:

$$\begin{aligned} b(t, -\infty) &= 1, \\ b(t, +\infty) &= 0, \\ n(t, -\infty) &= 0, \\ n(t, +\infty) &= 1. \end{aligned} \tag{S14}$$

Here, only the first equation may require an explanation. For a traveling front, the amount of nutrient consumed per unit of time in the entire system is $vn_0 = v$ since $n_0 = 1$ after nondimensionalization. Indeed, $\frac{d}{dt}\int n dx = -v\int n' dx = -v(1-0)$. Since all of this nutrient is converted into biomass, the rate of biomass production across the entire system must also equal v. Thus, $v = \frac{d}{dt}\int b dx = -v\int b' dx = -v(0 - b(t, -\infty))$ or equivalently $b(t, -\infty) = 1$.

Since the nutrient concentration at the colony edge is finite, we expect the traveling front to have the same asymptotic behavior at the colony edge as the models described by Eqs. (2) and (3) of the main text. Specifically, we anticipate that $b(z) = 0$ for $z \geq 0$ assuming that $z = 0$ at the colony edge [9, 40, 42]. Therefore, there are three important regions to analyze: $z > 0$, negative z near the colony edge, and negative z far away from the edge.

In the rightmost region with $b = 0$, the biomass equation is satisfied for any value of n, and the nutrient equation becomes independent of b and is easily solved. The nutrient profile is given by

$$n(z) = 1 - (1 - n_e)e^{-vz}, \tag{S15}$$

where $n_e = n(0)$ is the nutrient concentration at the edge of the colony that needs to be determined.

Within the colony, the nutrient concentration is still given by Eq. (S15) in the immediate vicinity of $z = 0$ because $n(z)$ is continuously differentiable. To determine the behavior of $b(z)$, we use the ansatz from Ref. [9], which is equivalent to the dominant balance of the first two terms in the equation (i.e., we neglect the last term because it does not contain derivatives, which are large at the front). Specifically, we look for $b(z) = -Az$, where A is a positive constant that can be determined upon the substitution of the ansatz into the equation:

$$DA^2(n + zn') + vA + Azn = 0. \tag{S16}$$

In the limit of $z \to -0$, we find that $A = -v/(Dn_e)$, and

$$b(z) = -\frac{v}{Dn_e}z. \tag{S17}$$

Finally, for large negative z, we first solve the equation for n by assuming that $b \approx 1$, and then solve the equation for b by assuming that n, b', and b'' are all small; in fact, they are of the same order due to the exponential approach to the boundary conditions. The linear equation for the nutrient is solved by the standard method and the solution reads

$$n = Ce^{\lambda z}, \tag{S18}$$

where C is a yet unknown constant, and λ is given by

$$\lambda = \frac{\sqrt{v^2 + 4} - v}{2}. \tag{S19}$$

The biomass equation can be written as

$$D(nb'^2 + bb'n' + bnb'') + vb' + bn = 0, \tag{S20}$$

and it is clear that all the terms with D are higher order than the remaining two terms. Upon further approximating b by 1, we find

$$b' = -\frac{n}{v} = -\frac{C}{v}e^{\lambda z}, \tag{S21}$$

which can be integrated to give the biomass profile

$$b = 1 - \frac{C}{v\lambda}e^{\lambda z}. \tag{S22}$$

This completes the analysis necessary to set up the shooting method. We integrate Eq. (S13) starting from an arbitrary point z_i with $n(z_i)$ and its derivative determined from Eq. (S18) and b and its derivative determined from Eq. (S22). Note that C must be chosen such that $n(z_i) \ll 1$. The integration stops when $b = 0$ or the solution becomes unphysical. At this final point, we have a numerically computed value of n_e, but the solution must also satisfy Eqs. (S15). This condition is not satisfied for an arbitrary v, so we perform a search to find v that results in a feasible solution.

Analytical approximation

We can obtain an approximate analytical solution for the traveling front profile and velocity by matching the solutions

at large negative z and at $z = 0$. That is, Eq. (S22) is matched with Eq. (S17), and Eq. (S18) is matched with Eq. (S15). This results in four conditions for b, b', n, and n':

$$\begin{aligned}
&1 - \frac{C}{v\lambda} = 0, \\
&-\frac{C}{v} = -\frac{v}{Dn_e}, \\
&C = n_e, \\
&C\lambda = v(1 - n_e).
\end{aligned} \tag{S23}$$

Upon eliminating C and n_e, we are left with two conditions:

$$\begin{aligned}
&\lambda^2 D = 1, \\
&\lambda^2 + \lambda v - 1 = 0.
\end{aligned} \tag{S24}$$

The second condition is exactly the same condition as Eq. (S19), so we have a unique solution for $v(D)$ and other parameters:

$$\begin{aligned}
&v = \sqrt{D} - \frac{1}{\sqrt{D}}, \\
&n_e = 1 - \frac{1}{D}, \\
&C = 1 - \frac{1}{D}, \\
&\lambda = \frac{1}{\sqrt{D}}.
\end{aligned} \tag{S25}$$

The expression for v is the results stated in Eq. (6) in the main text. Note that, if we restore the dimensional units, we need to multiply v by $\sqrt{D_n \gamma n_0}$. In the limit of large D, this gives $v = \sqrt{D_b \gamma n_0^3}$, which means that the nutrient diffusion constant does not affect the velocity in the large v limit.

Diffusion-limited growth

Numerical simulations show that, for $D < D_c$, the biomass profile approaches a step function moving outward as $t^{1/2}$. Moreover, the nutrient concentration at the edge of the colony drops to nearly zero, suggesting that the nutrients are immediately consumed when they reach the colony; see Fig. S6. In this section, we analyze this limit analytically.

Unlike for the traveling front solutions, the growth dynamics depends on the geometry of the problem even in the long time limit. Indeed, both the spatial extent of nutrient depletion and the radius of the colony grow as $t^{1/2}$, so their ratio stays constant and the growth dynamics do not reduce to the planar geometry. In the following, we assume that the growth instability is suppressed, e.g. by strong surface tension, and analyze spherically symmetric growth in d spatial dimensions. Thus, $d = 1$ corresponds to planar fronts, which could be realized in narrow channels, $d = 2$ corresponds to regular colonies grown on the surface of a Petri dish, and $d = 3$ corresponds to spherical aggregates akin early stage tumors.

We start with the following set of equations, which describe the asymptotic dynamics in the diffusion-limited regime:

$$\begin{aligned}
&\frac{\partial n}{\partial t} = \frac{\partial^2 n}{\partial r^2} + \frac{d-1}{r}\frac{\partial n}{\partial r}, \\
&H\frac{dr_e}{dt} = \left.\frac{\partial n}{\partial r}\right|_{r=r_e}, \\
&n(t, r_e) = 0, \\
&n(t, +\infty) = 1,
\end{aligned} \tag{S26}$$

where we used r to denote the distance from the colony center in any number of dimensions. When we are explicitly considering an one-dimensional situation, we revert to using x as the spatial coordinate. The subscript e refers to the colony edge.

Given that these equations describe diffusive dynamics, it is natural to seek the solution in the following form:

$$\begin{aligned} r_e(t) &= 2\varkappa\sqrt{t}, \\ n(t,r) &= n(\zeta), \\ \zeta &= \frac{r}{2\sqrt{t}}, \\ n(\zeta = \varkappa) &= 0, \\ n(\zeta = +\infty) &= 1, \end{aligned} \tag{S27}$$

where the fourth equation follows from $n(t, r_e) = 0$ and the ansatz for $r_e(t)$.

Upon replacing $n(t, r)$ by $n(\zeta)$, we find

$$\frac{d^2 n}{d\zeta^2} + \left(2\zeta + \frac{d-1}{\zeta}\right)\frac{dn}{d\zeta} = 0, \tag{S28}$$

which can be easily solved:

$$n = \frac{\int_\varkappa^\zeta dp p^{1-d} e^{-p^2}}{\int_\varkappa^{+\infty} dp p^{1-d} e^{-p^2}} = 1 - \frac{K_d(\zeta)}{K_d(\varkappa)}, \tag{S29}$$

where

$$K_d(y) = \int_y^{+\infty} dp p^{1-d} e^{-p^2}. \tag{S30}$$

Note that K_d is related to the upper incomplete gamma function, but we do not find that this connection is worth exploiting and instead specifically state K_d for one, two, and three dimensions below:

$$\begin{aligned} K_1(y) &= \frac{\sqrt{\pi}}{2}\operatorname{erfc}(y), \\ K_2(y) &= \frac{1}{2}\mathrm{E}_1(y^2) = \frac{1}{2}\int_{y^2}^{+\infty} \frac{e^{-q}}{q} dq, \\ K_3(y) &= \frac{e^{-y^2}}{y} - \sqrt{\pi}\operatorname{erfc}(y), \end{aligned} \tag{S31}$$

where $\operatorname{erfc}(y)$ and $\mathrm{E}_1(\mathrm{y})$ are the complimentary error function and exponential integral respectively.

From Eq. (S29), we can determine the nutrient flux at r_e and thus determine $\frac{dr_e}{dt}$:

$$H\frac{dr_e}{dt} = \left.\frac{dn}{d\zeta}\right|_{\zeta=\varkappa} \frac{\partial \zeta}{\partial r}. \tag{S32}$$

We can also determine $\frac{dr_e}{dt}$ by differentiating $r_e = 2\varkappa\sqrt{t}$ and thus obtain the following self-consistency condition:

$$H^{-1} = 2\varkappa^d e^{\varkappa^2} \int_\varkappa^{+\infty} p^{1-d} e^{-p^2} dp = 2\varkappa^d e^{\varkappa^2} K_d(\varkappa), \tag{S33}$$

which is Eq. (10) in the main text. This completes the solution of Eq. (S26) because we have determined the functional forms of $x_e(t)$ and $n(t, x)$ in terms of $\varkappa$, and found an implicit equation for $\varkappa$ in terms of H, which is a parameter in the simplified model.

The implicit equation for $\varkappa(H)$ can be easily analyzed in the limit of small and large $\varkappa$ by expanding $K_d(y)$ for small and large values of its argument. For slow expansions with $\varkappa \ll 1$, we find

$$\begin{aligned} H^{-1} &= \sqrt{\pi}\varkappa, \quad d=1, \\ H^{-1} &= -2\varkappa^2 \ln \varkappa, \quad d=2, \\ H^{-1} &= 2\varkappa^2, \quad d=3, \\ H^{-1} &= \frac{2\varkappa^2}{d-2}, \quad d>2, \end{aligned} \tag{S34}$$

which can be inverted to find $\varkappa(H)$:

$$\begin{aligned} \varkappa &= \frac{1}{\sqrt{\pi}H} = \sqrt{\frac{D}{\pi}}, \quad d=1, \\ \varkappa &= \frac{1}{\sqrt{H\ln(H)}} \approx \frac{D^{1/4}}{\sqrt{\ln(D)/2}}, \quad d=2, \\ \varkappa &= \frac{1}{\sqrt{2H}} = \frac{D^{1/4}}{\sqrt{2}}, \quad d=3, \\ \varkappa &= \sqrt{\frac{d-2}{2H}} = D^{1/4}\sqrt{\frac{d-2}{2}}, \quad d>2, \end{aligned} \tag{S35}$$

where we used the result that $H = 1/\sqrt{D}$, which is derived below. Note that for $d=2$ the asymptotic expression given above might be better approximated by the exact solution of $H^{-1} = -2\varkappa^2 \ln \varkappa$, which reads $\varkappa = \exp[\mathrm{W}_{-1}(-H^{-1})/2]$, where $\mathrm{W}_{-1}(\cdot)$ is the Lambert W function. Note that for a given value of H or D, the expansion rate $\varkappa$ increases with d, with the biggest difference between $d=1$ and $d=2$. This speed up reflects the increasing ratio of the surface to the volume in higher dimensions, i.e. the greater amount of nutrient that can diffuse from the outside in a given solid angle.

To restore the dimensional units, we need to multiply the dimensionless value of $\varkappa$ by $\sqrt{D_n}$. In one dimension, we find that $\varkappa = n_0\sqrt{D_b/\pi}$ for small D. Thus, the rate of the colony expansion is independent of D_n even though the growth is diffusion limited. In two dimensions, we find that, for small D, $\varkappa = D_n^{1/4}D_b^{1/4}n_0^{1/2}/\sqrt{\ln(D_b n_0^2/D_n)/2}$, so both D_n and D_b affect the rate of colony growth. Experimentally, the easiest quantity to vary is n_0, and our results predict that $\varkappa \propto n_0$ in narrow channels while $\varkappa \propto \sqrt{n_0}$ for circular colonies (up to logarithmic corrections). Thus, the growth geometry controls not only the rate of colony growth, but also its dependence on the nutrient concentration. Note that the scaling of the traveling front velocity with n_0 is different from that of $\varkappa$. For $D \gg 1$, we expect that $v \propto n_0^{3/2}$.

In addition to the rate of square-root expansion $\varkappa$, we can characterize the growth of the colony by the rate of biomass accumulation $B(t) = S_d H(2\varkappa\sqrt{t})^d$, where S_d is the area of unit sphere embedded in d dimensions. Using our results above we find that

$$\begin{aligned} B(t) &= \frac{2t^{1/2}}{\sqrt{\pi}}, \quad d=1, \\ B(t) &= \frac{8\pi t}{\ln(H)} = -16\pi t\frac{1}{\ln(D)}, \quad d=2, \\ B(t) &= \frac{16\pi t^{3/2}}{\sqrt{2H}} = 8\sqrt{2}\pi t^{3/2}D^{1/4}, \quad d=3, \\ B(t) &= S_d 2^d t^{d/2} H^{\frac{2-d}{2}}\left(\frac{d-2}{2}\right)^{d/2} = S_d 2^d t^{d/2} D^{\frac{d-2}{4}}\left(\frac{d-2}{2}\right)^{d/2}, \quad d>2. \end{aligned} \tag{S36}$$

For $d=1$, we can set $D=0$ and observe that our expression for $B(t)$ matches the expected amount of nutrient absorbed by a stationary colony. (In dimensional units, $B(t) = 2n_0\sqrt{D_n t/\pi}$; note, H scales as n_0 not as distance.) Therefore, a small increase in D above zero produces only moderate increase in $B(t)$ compared to a stationary colony.

For large $\varkappa$, however, a moving colony consumes much more nutrient, and higher biomass motility D is greatly beneficial for any d; see Eq. (S38).

For $d = 2$, $B(t) = -16\pi n_0 D_n t / \ln(D_b n_0^2/D_n)$ in dimensional units. The predicted logarithmic dependence on D_b, however, could be influenced by long transients due to the competition between the solution with small, but nonzero D_b and the solution for a stationary colony of a nonzero radius, which also leads to $B(t) \propto t$. In fact, large inoculations, could produce colonies for which the latter solution dominates. Small inoculations, e.g. started from a few cells, should however be described by our solution provided D_b is not too small.

For $d = 3$, our results may not be directly applicable to actual growing populations, at least in the limit of $H \to +\infty$, because H corresponds to the amount of biomass packed inside physical space. For $d = 1$ and $d = 2$, the biomass can escape in the third dimension, but this is not possible for $d = 3$, where H corresponds to biomass compression. Given that cell are nearly incompressible, we do not expect that three dimensional aggregates can achieve large values of H, and their motility should be described by the pressure-driven model of an incompressible fluid.

For fast expansions with $\varkappa \gg 1$, which occur for $H \to 1$ and $D \to 1$, Eq. (S33) yields the following asymptotic results:

$$H^{-1} = 1 - \frac{d}{2\varkappa^2}, \tag{S37}$$

which can be inverted to find $\varkappa(H)$:

$$\varkappa = \sqrt{\frac{Hd}{2(H-1)}} = \sqrt{\frac{d}{2(1-\sqrt{D})}}, \tag{S38}$$

where we again used the result that $H = 1/\sqrt{D}$. As for $\varkappa \ll 1$, we find that, for $\varkappa \gg 1$, colonies expand faster in higher dimensions, but the speed up from $d = 1$ to $d = 2$ is less dramatic. More importantly, we find that the rate of the square-root expansion diverges as H approaches unity from above. These results are easy to understand: The bigger the H, the more nutrient is necessary to advance the colony edge forward. Therefore, thick colonies put most of their biomass growth in the vertical direction and expand slowly, while thin colonies put all of their growth into the horizontal direction and expand faster. This feedback between local biomass accumulation and outward expansion is nonlinear because the faster the outward expansion, the greater the amount of nutrient that the colony consumes. Equation (S33) captures this nonlinear feedback quantitatively.

Derivation of Eq. (11)

Figures 1 and 3 in the main text provide strong evidence that the simplified model defined by Eq. (S26) indeed describes the long-time behavior of Eq. (S12). The only missing link in this correspondence is the dependence of H on D, which are the sole parameters in the respective models. Intuitively, we expect that larger D should result in greater $\varkappa$ and, therefore, lower H. Here, we provide an approximate derivation of $H(D)$, which is stated as Eq. (11) in the main text.

As we mentioned above, the biomass profile remains sharp, i.e, it has a constant width, even though the region of nutrient depletion grows as $t^{1/2}$. Therefore, we can neglect the curvature of the biomass front. We show below that the nurient equation also reduces to the plane-wave geometry, and, therefore, Eq. (11) holds for any d. Our approach is to follow the steps of the previous section on the traveling-front solutions, with two important differences. First, the biomass density behind the front now saturates at H instead of 1. Second, we now treat $n_e = n(t, r_e(t))$ and $v = \frac{dr_e}{dt} = \frac{\varkappa}{\sqrt{t}}$ as slowly varying functions of time. That is, we do not neglect their time dependence, but we do neglect their time derivatives because they vanish much faster, as $t^{-3/2}$, at long times.

We start with the nutrient profile, which we assume is of the form $n_e(t)\tilde{n}(z)$, where $z = r - r_e(t)$. For $r > r_e$, Eq. (S29) provides a good approximation to $n(t, r)$. However, since by assumption $n_e(t) = 0$, we cannot use this equation directly. Instead, we compute the flux of the nutrient because it does not vanish and therefore could be used to link the solutions for $r > r_e$ and $r < r_e$. This flux is given by $H\frac{dr_e}{dt} = \frac{H\varkappa}{\sqrt{t}}$, so we require that

$$\left.\frac{\partial n}{\partial r}\right|_{r=r_e} = \frac{H\varkappa}{\sqrt{t}}. \tag{S39}$$

Inside the colony, the nutrient profile approximately satisfies Eq. (S13) with the modifications specified above:

$$\frac{d^2\tilde{n}}{dz^2} + \frac{d-1}{r}\frac{d\tilde{n}}{dz} + v(t)\frac{d\tilde{n}}{dz} - H\tilde{n} = 0. \tag{S40}$$

Note that both $v(t)$ and r_e^{-1} decrease as $t^{-1/2}$, and a nontrivial limit exists even when $v = 0$ and $r \to +\infty$:

$$\frac{d^2\tilde{n}}{dz^2} - H\tilde{n} = 0. \tag{S41}$$

Thus, to the leading order, we find that $\tilde{n} = e^{\sqrt{H}z}$, and, therefore,

$$n(t, r) = n_e(t)e^{\sqrt{H}(r - r_e(t))}. \tag{S42}$$

Upon combining Eqs. (S39) and (S42), we obtain the nutrient concentration at the colony edge:

$$n_e(t) = \frac{\sqrt{H}\varkappa}{\sqrt{t}}. \tag{S43}$$

Next, we turn to the biomass profile, $b(t, r) = b(r - r_e(t))$. Near the colony edge, all the steps leading to Eq. (S17) remain valid, and we immediately obtain that

$$b(z) = -\frac{v(t)}{Dn_e(t)}z = -\frac{1}{D\sqrt{H}}z. \tag{S44}$$

Note that the time dependence of v and n_e cancels out, so the shape of $b(z)$ has no time dependence, in agreement with simulations.

The final equation that we need is Eq. (S20), which we use to obtain the biomass profile for large negative z. This equation also remains unchanged given our assumption of slowly varying $v(t)$ and large r. As before, we neglect the first three terms that contain two spatial derivatives because they are much smaller than the remaining terms, given than both n and b approach their limiting values exponentially in z. We then conclude that

$$b' = -\frac{bn}{v} = -\frac{Hn_e(t)\tilde{n}(z)}{v(t)} = -H^{3/2}\tilde{n}(z), \tag{S45}$$

where the time dependence again cancels out.

Similar to our approximation for traveling fronts, we now match the behavior of b for large and small z, i.e., we match Eqs. (S44) and (S45). This gives

$$\frac{1}{D\sqrt{H}} = H^{3/2}, \tag{S46}$$

or equivalently

$$H = \frac{1}{\sqrt{D}}, \tag{S47}$$

which is the same as Eq. (11) in the main text. The general dependence of $\varkappa$ on D follows from Eqs. (S47) and (S33) as discussed above.

Note that, if we restore the dimensional units of H then we need to multiply it by n_0 since H is a measure of biomass not distance. Hence, $H = \sqrt{D_n/D_b}$, i.e., it does not depend on n_0 and γ.

Simulations

Equation (S12) and other models were solved using a finite difference method. The biomass equation was solved using an explicit method, and the nutrient equation was solved using the Crank-Nicolson method [48]. All simulations were started from a small initial biomass concentration at the left edge of the simulation box, with uniform nutrient concentration, and were ran until the nutrients at the right edge of the simulation box started to be consumed. All the figures were obtained with a no-flux boundary condition for the biomass and a Dirichlet boundary condition for the nutrients. We also performed simulations with a no-flux boundary condition for the nutrients and the result are identical, since we stop the simulation as soon as boundary effects materialize. The simulation and analysis codes are available at `https://github.com/lbrezin/fronts-to-diffusion`.

Supporting figures

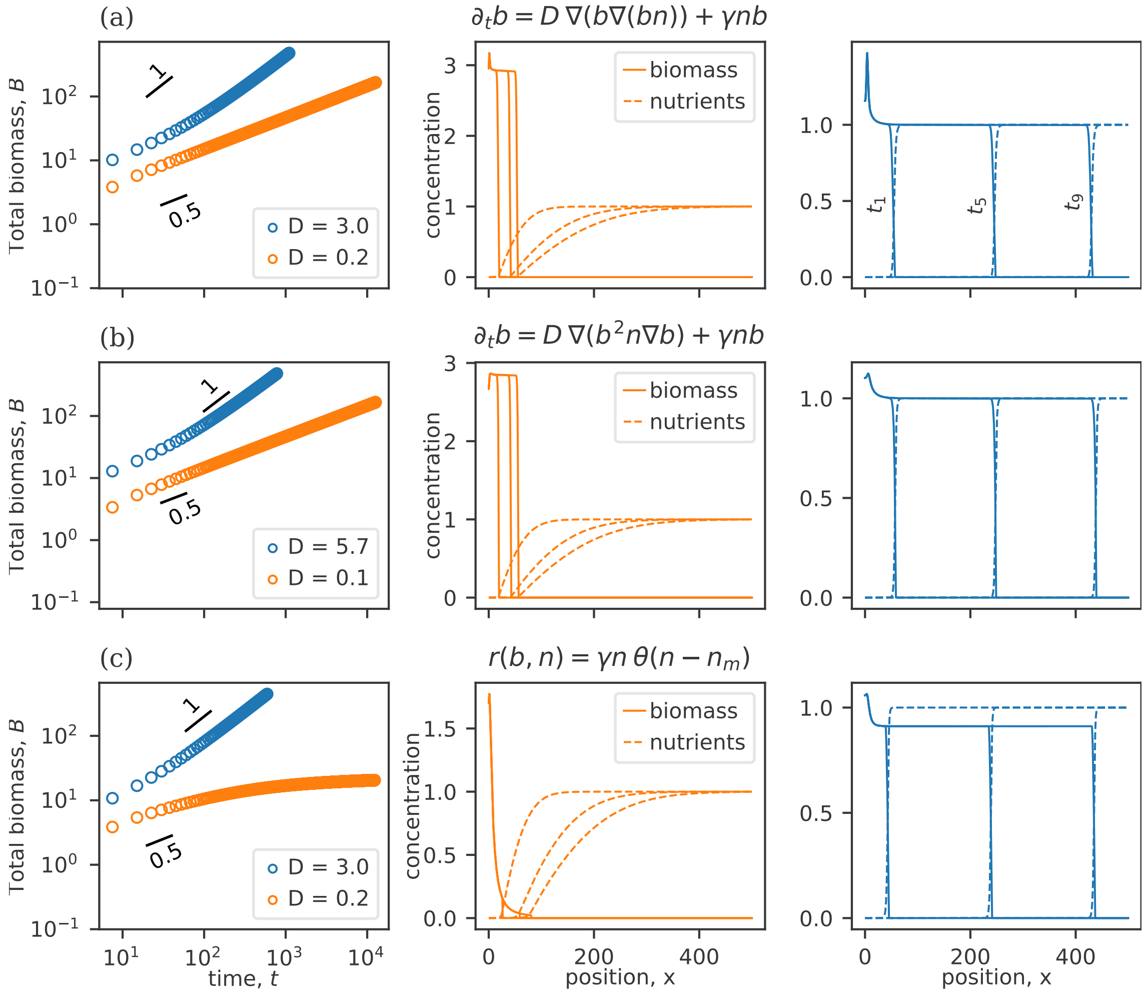

FIG. S1. Transition from traveling fronts to diffusion-limited growth is a general feature of nutrient-dependent diffusion. For different models, in 1 dimension, we show the increase of biomass over time at low and high rates of biomass redistribution (left column), and the corresponding biomass and nutrient profiles at 10%, 50%, and 90% of the time preceding nutrient depletion at the end of the simulation box. Models without a maintenance cost (rows (a) and (b)) exhibit both a linear and a square-root increase of the total biomass with time. Introducing a maintenance cost (row (c)) drastically changes the behavior at low dispersal. The nutrient concentration at the edge of the colony decreases over time until it drops below the maintenance cost threshold, at which point the biomass stops growing. For the traveling-front growth, the biomass never reaches the maximum value set by the initial concentration of nutrients because of the maintenance cost.

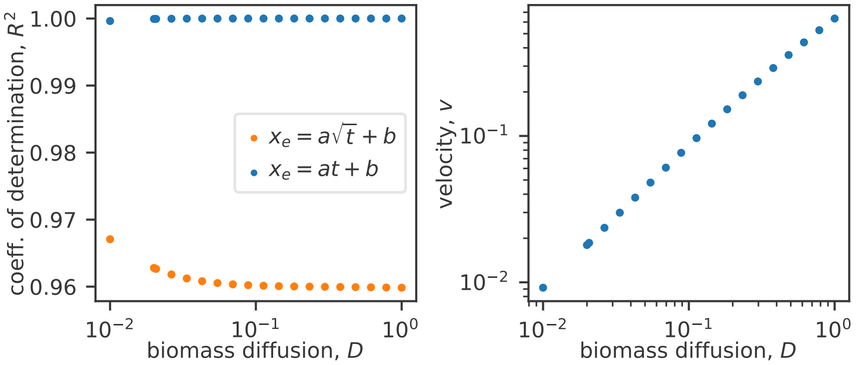

FIG. S2. The model introduced in Eq. (3) in the main text, with the motility independent of nutrient concentration, does not exhibit the transition seen in our model. There is a traveling wave solution at low D with a velocity that scales linearly with D for $D \ll 1$ [9]. The uptick seen at low D for the square-root fitting is due to rapidly increasing transient times as $D \to 0$.

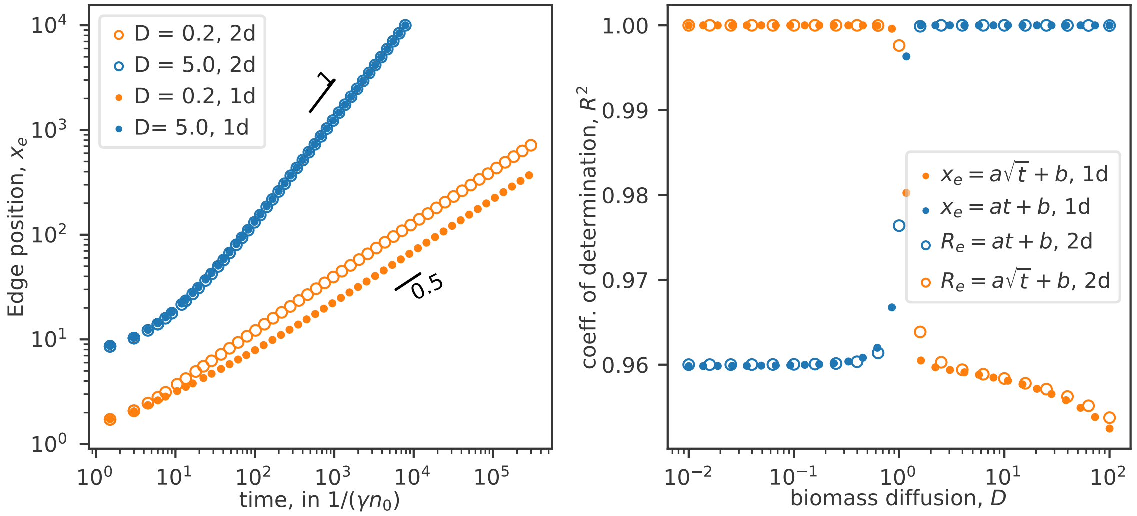

FIG. S3. Comparing simulations of Eq. (4) in 1 and 2 dimensions. The traveling wave solution is identical in both cases, and the transients are different in the diffusion-limited growth, but the transition remains the same at $D_c = 1$.

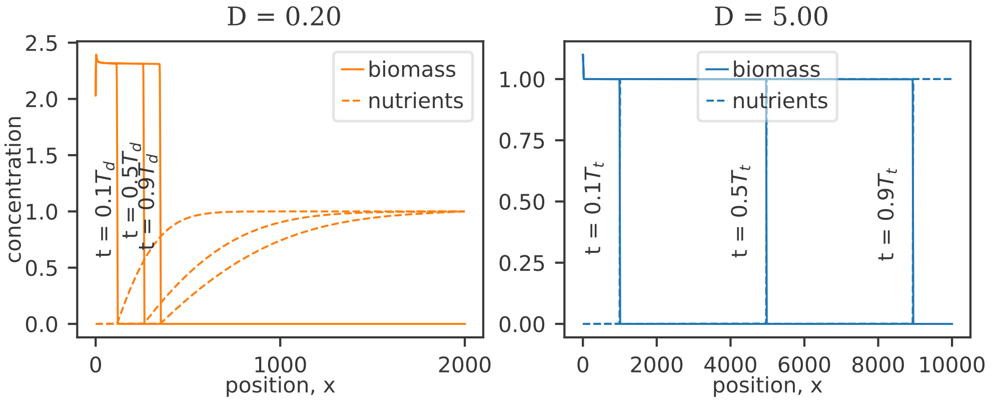

FIG. S4. Biomass and nutrient profiles in 1d for diffusion-limited growth and traveling front. The profiles are similar to the 2d case shown in the main text in Fig. 1c-d.

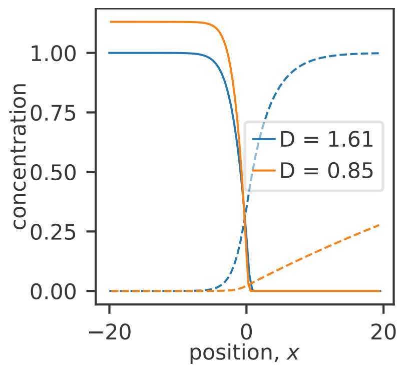

FIG. S5. Close-up view of the biomass and nutrient profiles near the edge of the colony, for a 1d expansion. For the traveling wave solution at $D > D_C$ (blue lines) the size of the depletion layer is comparable to the width of the biomass front. For the diffusion-limited growth solution at $D < D_C$ (yellow lines), the size of the depletion layer is much greater than the width of the biomass front

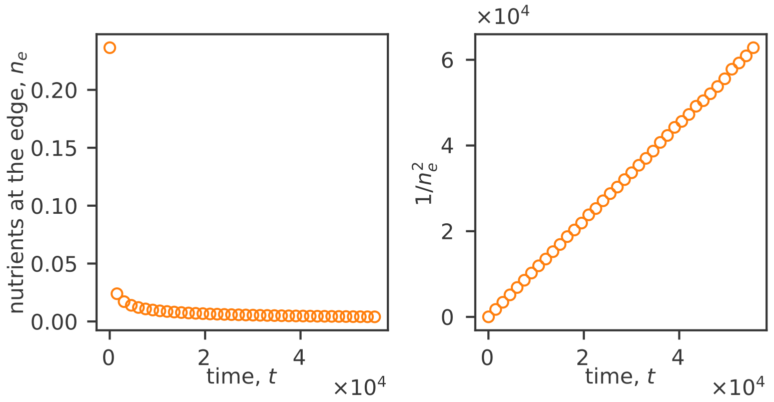

FIG. S6. The nutrient concentration at the colony edge vanishes an $t^{-1/2}$ as predicted by (S43).

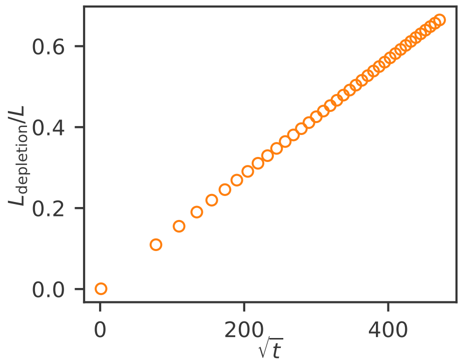

FIG. S7. The size of the depletion layer $L_{\mathrm{depletion}}$ increases as $t^{1/2}$. We see a linear relationship between $L_{\mathrm{depletion}}$ normalized by the size of the system as a function of the square root of time, for the diffusion-limited growth. We define $L_{\mathrm{depletion}}$ as the distance between the leading edge where the biomass is 5% of its maximal value and the first point where there is full nutrient availability (nutrients above 99% of their initial value).

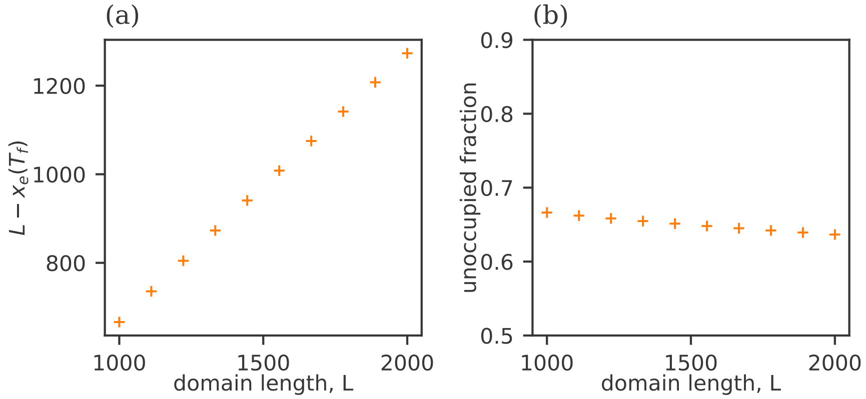

FIG. S8. The distance between the edge of the system L and the edge of the colony once all nutrients are depleted $x_e(T_f)$ is expected to increase as a function of the system size L in the diffusion-limited regime (a), while we expect the unoccupied fraction $\frac{L-x_e}{L}$ to stay roughly the same (b). Increasing system size is equivalent to increasing total simulation time, therefore increasing the size of the depletion layer until it reaches the boundary. For the fraction, both the colony edge position and the depletion layer increase as $t^{1/2}$, such that their ratio is expected to be independent of time. Therefore, we expect the unoccupied fraction to be constant.
